# Qatar steps up to Global Health security: a reflection on the joint external evaluation, 2016

**DOI:** 10.1186/s41256-017-0050-y

**Published:** 2017-10-18

**Authors:** Mohamed Osman Bala, Mohamad Abdelhalim Chehab, Nagah Abdel Aziz Selim

**Affiliations:** 1 Community Medicine Residency Program, Hamad Medical Corporation, Doha, Qatar; 2 Primary Health Care Corporation, Doha, Qatar; 3 Faculty of Medicine, Cairo University, Giza, Egypt

**Keywords:** Global health security, International health regulations, Joint external evaluation, Qatar

## Abstract

Since the commencement of the International Health Regulations in 2007, global public health security has been faced with numerous emerging and ongoing events. Moreover, the Joint External Evaluation is a voluntary tool developed in compliance with the Global Health Security Agenda that represents the high responsibility of international health community towards the increased incidence of emerging and re-emerging diseases. Against this background, between 29th May and 2nd June 2016, a team of World Health Organization consultants arrived to the State of Qatar to assess, in collaboration with national experts, the country’s capacity to prevent, detect, and rapidly respond to threats of public health aspect. They identified areas of strength, weakness, and recommendations for improving national health security of Qatar in anticipation of the 2022 FIFA World Cup event. Qatar has demonstrated a leading role in the region through its commitment to International Health Regulations (2005) and population health. Similarly, the Qatar was the first Arab state and seventh volunteering country globally to undergo the Joint External evaluation process. In this review, we highlighted Qatar’s achievements and shortcomings of International Health Regulations’ core capacities to inform healthcare professionals and the scientific community about the country’s contribution toward global health security.

## Background

Since the commencement of the International Health Regulations (IHR, 2005) in 2007, global public health has been faced with numerous emerging and ongoing events such as the avian and pandemic flu, cholera, Middle East Respiratory Syndrome (MERS) Coronavirus, Ebola, and recently the Zika virus epidemic. These events have hindered any progress in implementing core capacities of the prevention, detection, and adequate response to health emergencies. Moreover, the increasing rate and diversity of infectious disease threats jeopardizes the IHR accomplishments and hence the foundations of global health security.

Against this background, between 29th May and 2nd June 2016, a team of World Health Organization (WHO) consultants arrived to the State of Qatar to assess, in collaboration with national experts, the country’s capacity to prevent, detect, and rapidly respond to threats of public health aspect, whether natural, intentional, or inadvertent; in accordance with IHR 2005 [1]. The joint assessment was conducted using the WHO IHR (2005) - Joint External Evaluation (JEE) tool which is a data gathering instrument encompassing 19 technical areas categorized within three major components; prevention, detection and response. Every technical area has multiple questions that will help the evaluators determine the appropriate score [2].

The JEE tool was developed in compliance with the Global Health Security Agenda (GHSA) that represents a reaction by the international health community towards the increased incidence of emerging and re-emerging diseases (e.g. Ebola, Yellow Fever, avian influenza, MERS coronavirus) and the threat these pose on the global health security due to interconnectedness of today’s world [3]. Additionally, the GHSA aims at promoting the adherence and mobilization of the nations of the world behind the full implementation of the IHR as well as the World Organization of Animal Health’s (OIE) Performance of Veterinary Services Pathway, and similar health security frameworks through a multinational and multisectoral approach [4]. It was established and declared on February 2014 in the White House, as an initiative led by the United States of America with 44 committed countries to become a five-year plan focused on empowering public health capacities regarding human as well as animal infectious threats [5].

### The JEE process

The JEE process is a voluntary, collaborative process that is comprised of many steps. The first step involved the completion of Qatar’s country survey through self-reporting data on specific indicators. After which, this information is sent to the external evaluation team as well as subject matter specialists who will utilize this information to establish a baseline for Qatar’s health security capacity. The third step comprised the site visit by the evaluation team to Qatar, where in-depth discussions with the national experts as well as structured visits to vital ministries, healthcare institutions, and the points of entry were conducted to identify strengths, obstacles, opportunities, and priorities. Then, the team drafted a final report of the findings according to the predefined indicators and shared it with the State of Qatar and after the latter’s permission with the international stakeholders and community. Interestingly, the indicators evaluated the country’s capacity development on a score of 1 to 5 and were color coded, where 1 (color code red) signified the absence of capacity while 5 (color code green) described a sustainable capacity. Finally, the evaluation team is expected to conduct a consecutive visit to follow up on the findings and recommendations after approximately 5 years from the last visit [2].

### The results

The evaluation report classically presents the findings through the aforementioned three core components, which are prevention, detection, and response with an additional miscellaneous section (points of entry, chemical events, radiation emergencies). Within each of the aforementioned core components, the report reveals the strengths, the weaknesses, and recommendations for improvement based on the 19 technical areas. In general, Qatar has demonstrated variable levels of capacity in the different technical areas, with individual scores ranging between 2 (limited capacity) and 5 (sustainable capacity).

### Prevention

Within this component, the report encompasses many sections, such as: national legislation, policy and financing, IHR coordination, communication and advocacy, antimicrobial resistance, zoonotic diseases, food safety, biosafety and biosecurity, and immunization. The most critical of these areas was the antimicrobial resistance section, where Qatar showed limited capacity concerning the antimicrobial stewardship activities. However, Qatar has developed capacity (score:3) in most domains: antimicrobial resistance detection, surveillance of infections caused by antibiotic-resistant pathogens, and health care-associated infection prevention and control programs. In order to further build Qatar’s capacity, one of the best recommended practices to fight antimicrobial resistance is the One Health approach. The approach includes an integrated global package of activities to combat antimicrobial resistance across human, agricultural, food, animal, and environmental aspects; which was developed in coordination between the WHO, Food and Agriculture Organization (FAO), and OIE [6]. This practice has been successfully adopted by Qatar in response to MERS ongoing outbreaks [7].

Interestingly, Qatar has established a legal framework to allow the implementation of the IHR through enabling laws and regulations; thus, it has established capacity (score: 4) with this regards. However, there are recommendations for further improvement through reactivating a previously established IHR national committee, developing a national framework law and bylaws in a unified official document, and updating decrees as well as laws to enable further IHR implementation.

**Fig. 1:**
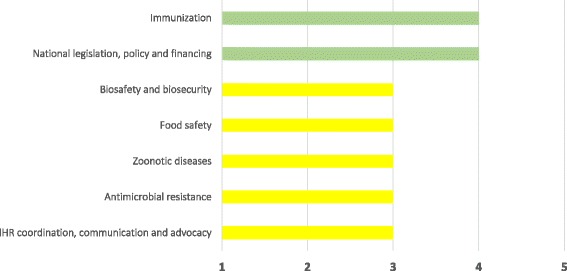
Score of Qatar against the IHR Joint External Evaluation: Prevention component 1 = No capacity, 2 = Limited capacity, 3 = Developed capacity, 4 = Demonstrated capacity, 5 = Sustainable capacity Data reproducible from JEE report (http://apps.who.int/iris/bitstream/10665/254509/1/WHO-WHE-CPI-2017.6-eng.pdf?ua=1)

Moreover, Qatar has developed an immunization program characterized by a national coverage, effective cold chain and distribution, equitable access, and continuous quality control measures. Thus, the country attained a sustainable capacity of measles vaccine coverage as well as an established capacity of vaccine access and delivery. Also, Qatar is currently supporting other countries in the region with their attainment of national measles vaccine coverage as per IHR (2005). (Fig. 1).

### Detection

**Fig. 2:**
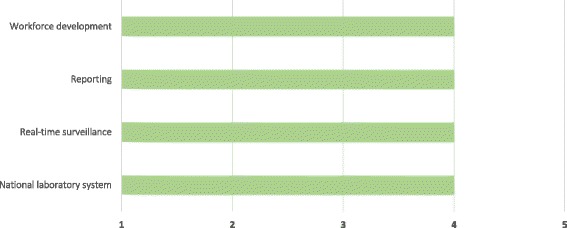
Score of Qatar against the IHR Joint External Evaluation: Detection component 1 = No capacity, 2 = Limited capacity, 3 = Developed capacity, 4 = Demonstrated capacity, 5 = Sustainable capacity Data reproducible from JEE report (http://apps.who.int/iris/bitstream/10665/254509/1/WHO-WHE-CPI-2017.6-eng.pdf?ua=1)

The detection aspect of the report included the following sections: national laboratory system, real-time surveillance, reporting, and workforce development. Qatar’s scores within this component manifest an established capacity in multiple technical areas; however, the system has only developed capacity (score 3) within the laboratory quality system, electronisation and interoperability of the reporting system, analysis of the surveillance data, national field epidemiology training program, and workforce strategy (Fig. 2).

The role of national laboratory system is emphasized by the JEE; where this role is a significant prerequisite for establishing real-time biosurveillance and delivering effective modern point-of-care as well as laboratory-based diagnostics. In addition, the evaluation has revealed an urgent need for better coordination of all laboratories at a central level including the medical, veterinary, and environmental sectors.

Regarding the reporting system and surveillance data analysis, IHR and OIE standards demand complementary and synergistic indicator as well as event-based surveillance systems. Qatar currently has a 67 notifiable disease list, relying mainly on indicator-based surveillance, where data is collected and input manually due to the absence of an electronic system. The aforementioned goal requires further capacity building through recruitment of new staff as well as further training of those retained, approval and commencement of the new proposed communicable disease control law, establishment of guiding documents (e.g. guidelines, standard operating procedures, case definitions), and applying the one health approach to the surveillance system. Importantly, the surveillance system should engage the stakeholder through sharing relevant reports and feedback. In addition, the system must undergo routine comprehensive evaluation for its development.

As comes to national field epidemiology and the workforce strategy, the Arab Board Community Medicine Residency Program (4 years) is the only locally available program to provide some field training in Qatar. Thus, establishing a dedicated surveillance and outbreak response training program is recommended; along with a human resource strategy to formulate a clear career pathway and in return allow for the engagement and retaining of homegrown as well as international experts.

### Response

**Fig. 3:**
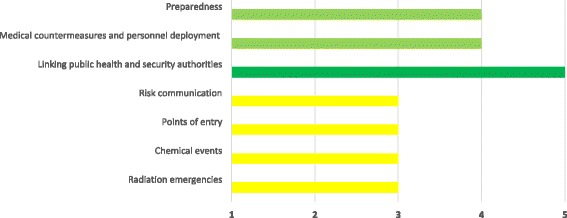
Score of Qatar against the IHR Joint External Evaluation: Response and others components 1 = No capacity, 2 = Limited capacity, 3 = Developed capacity, 4 = Demonstrated capacity, 5 = Sustainable capacity Data reproducible from JEE report (http://apps.who.int/iris/bitstream/10665/254509/1/WHO-WHE-CPI-2017.6-eng.pdf?ua=1)

Regarding the response component, the report elaborated on the following issue: preparedness, emergency response operations, linking public health and security authorities, medical countermeasures and personnel deployment, and risk communication. Overall, the country has demonstrated good performance within the emergency preparedness and response subsections; where score ranged mostly between 4 and 5. However, it seems that Qatar has demonstrated but not yet established capacity with regards to risk communication, especially the communication systems, intra- and intersectoral coordination, public communication, engaging the affected communities, and rumor management (Fig. 3). Thus, to develop a comprehensive national risk communication system, there is need for a health sector risk communication strategy, a dedicated risk communication unit at the central level, i.e. Ministry of Public Health, to support the above strategy, mock risk communication emergency exercises within the health sector and nationwide, identification of the target audience in Qatar through maps (nationals and expatriates),and strengthening community engagement activities through staff training as well as research and certified course for community volunteers.

### Others

The others’ part of the report involved information about Qatar’s points of entry, chemical events, and radiation emergencies. In the report, the State of Qatar demonstrated a developed capacity (score:3) in the related six technical areas with regards to capacities and public health response at the points of entry as well as the mechanisms and enabling environment for managing chemical, radiological, and nuclear events or emergencies (Fig. 3).

Qatar has six recognized points of entry that support international traffic and four seaports which are the followings:

- Doha Port (Mwani Ports), used by general cargo ships and container vessels;
- Mesaieed Port (Qatar Petroleum), used by vessels in the oil/gas sector together with general cargo and container ships delivering goods to the designated Mesaieed industrial area;
- Ras Laffan Port (Qatar Petroleum), used by vessels in the oil/gas sector;
- Al Ruwais seaport, used by dhow-type vessels for smaller cargo trade;
- Hamad International Airport, a major international airport and the hub airport of Qatar Airways;
- Abu Samra ground crossing, the main land entry point to Qatar at the border with Saudi Arabia

This reflects recent developments in the transport sector all over the country. One of the aforementioned JEE components was that core capacities and potential hazards management should be applied at the points of entry. As a result, it was revealed that Qatar has a developed capacity in most technical indicators; despite some deficiencies reported in the vector control program as well as the surveillance and inspection program of vessels for sanitation purposes, especially at seaports’ points of entry.

Multiple stakeholders are responsible for the chemical safety in Qatar, including the ministries of interior, defense, industry, public health, and municipality and environment as well as Qatar Petroleum. To further develop this sector and fortify national health security, it was recommended to establish channels for sharing information across the aforementioned stakeholders, develop a surveillance system for chemical incidents, and enable laboratory detection and analysis of chemicals hazardous to human and environmental health.

## Conclusion

Qatar has demonstrated a leading role in the region through its commitment to International Health Regulations (2005) and community. Similarly, Qatar was the first Arab state and seventh volunteering country globally to undergo the JEE process. In anticipation of the 2022 World Cup event planned in Qatar, the country has a golden opportunity to fortify its capabilities and rectify the weaknesses of its health security system.

## Abbreviations

GHSA: Global Health security agenda
IHR: International Health regulation
JEE: Joint external evaluation
MERS: Middle East respiratory syndrome
OIE: World Organization of Animal Health
